# Bilateral giant emphysematous bullae complicate severe bronchial asthma in a young patient: case report

**DOI:** 10.1093/jscr/rjad470

**Published:** 2023-08-14

**Authors:** Novath Ngowi, Maurice Mavura, Catherine Martin

**Affiliations:** Department of Surgery, Muhimbili University of Health and Allied Sciences (MUHAS), Dar es Salaam, Tanzania; Department of General Surgery, Muhimbili National Hospital (MNH), Dar es Salaam, Tanzania; Department of Surgery, Muhimbili University of Health and Allied Sciences (MUHAS), Dar es Salaam, Tanzania

**Keywords:** emphysematous bullae, bullectomy, asthma

## Abstract

Giant bullous emphysema is a progressive bullous disease that affects young male smokers. Bullae are unilateral and mostly present in the apical lobes. Inflammatory diseases are less common cause of underlying emphysematous deterioration of the lung than tobacco smoking or genetic conditions such as Alpha-1 antitrypsin deficiency. The current instance, however, is relatively rare because it involved a nonsmoking 14-year-old boy who was diagnosed with asthma for 8 years, and he was taking bronchodilators inhalers during acute exacerbation of asthma; he presented to the tertiary health facility with on-and-off episodes of difficulties in breathing and chest tightness for 2 weeks despite being on maximal therapy for his asthma. He was diagnosed with bilateral large emphysematous bullae by high-resolution computed tomography scan, where staged bilateral bullectomy was performed. Thoracotomy-based bullae excision is still a feasible option for improving pulmonary function and the overall quality of life of patients with giant bullae emphysema in resource-limited settings.

## INTRODUCTION

Bronchial asthma is a clinical illness marked by reversible airway obstruction, produced by enhanced airway hyper-responsiveness to numerous stimuli, which can be resolved with treatment [1]. An asthma exacerbation can be divided into two stages: the early phase and the late phase. IgE antibodies, which are sensitized and secreted by plasma cells, start the early phase. These antibodies react to environmental cues; IgE antibodies then attach to mast cells and basophils with high affinity. Mast cells produce cytokines and eventually de-granulate when a pollutant or risk factor is ingested. Histamine, prostaglandins and leukotrienes are all released by mast cells. These cells then contract the smooth muscle, narrowing the airway [2].

Bullous emphysema is characterized by the presence of centrilobular emphysema in the non-bullous lung in a patient with chronic obstructive pulmonary disease (COPD). Bullous lung disease differs from bullous emphysema in that bullae are associated with structurally sound intervening lungs, whereas bullous emphysema is linked with bullae associated with more diffusely defective lung parenchyma due to COPD [3]. Giant bullous emphysema is characterized by massive bullae that encompass at least one-third of one or both hemithorax and typically affect young male smokers.

For more than a century, surgical management has attempted to improve pulmonary function and health-related quality of life for patients with advanced emphysema. Only giant bullectomy, lung transplantation and lung volume reduction surgery (LVRS) have withstood the test of time and are currently in use. Emphysema with damage and hyperinflation, severe impairment (FEV1 35% predicted), significant restriction in daily activities and failure of maximal therapy to alleviate symptoms are all common indications for lung transplantation and LVRS procedures [4].

## PATIENT AND OBSERVATION

A 14-year-old boy, who had been diagnosed with asthma for 8 years and had been taking salbutamol, budesonide and Montelukast during episodes of acute exacerbation of asthma, presented to our institution with on-and-off episodes of difficulty in breathing and chest tightness for 2 weeks despite using bronchodilators. It began gradually but became increasingly severe over time, accompanied by a whistling sound, cough during night and morning time which was exacerbated by strenuous activities; it was productive with whitish sputum. However, he had no constitutional symptoms of tuberculosis (TB). Over the course of his current illness, the patient had received treatment from multiple primary health facilities, including oxygen therapy and nebulized with salbutamol, without relief till when he was referred to the Muhimbili National Hospital for further management. He had several similar episodes over the past 8 years; he was admitted in our hospital and received oral and intravenous medications and the symptoms subsided.

The patient received childhood vaccinations, including BCG, OPV, Pneumococcal, influenza, Rota and measles. He had history of pulmonary tuberculosis (PTB), underwent tonsillectomy in 2020 and recurrent URTIs. He had allergies to certain medications, specifically NSAIDs.

### Patient observation

**Figure 1:**
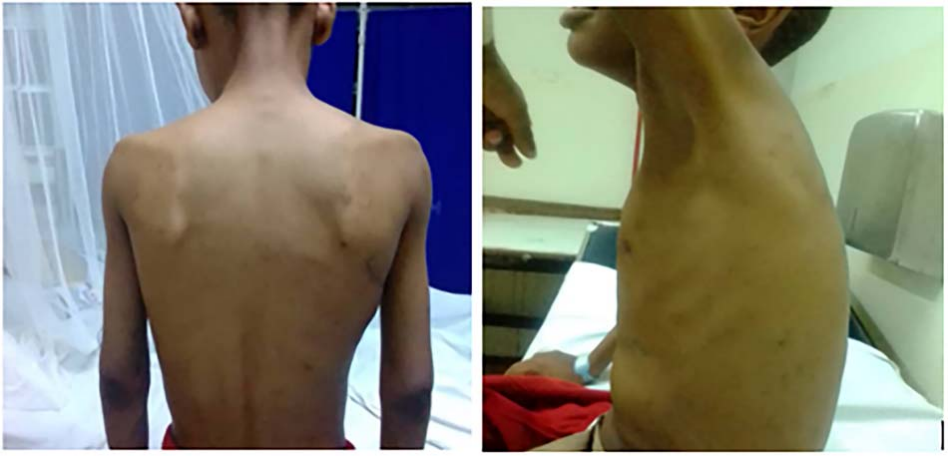
Barrel shape of the chest with scoliosis.

The patient was admitted with a fever, dyspnea and a fever of 370°C. He had high blood pressure, a respiratory rate of 23 breaths/min and 95% oxygen saturation. He had 26 kg weight and a body mass index of 15.4 kg/m^2^. His respiratory examination showed a barrel shape with scoliosis, symmetrical chest movement, no surgical or therapeutic marks and a centrally located trachea, with hype-resonant on percussion, reduced breath sounds more on the right with a wheezing sound (Fig. 1).

He was nebulized with salbutamol (2.5 mg) and budesonide (0.25 mg) through NRBM with oxygen 15 l/min, iv dexamethasone 12.5 mg start, magnesium sulfate intravenous infusion 2 g/h (50 ml/h of 20 g/500 ml) on arrival at the emergence department. During admission, blood workup and radiological investigation were done; initially, CXR was done and then HRCT of the chest was done.

### Diagnostic assessment

**Figure 2:**
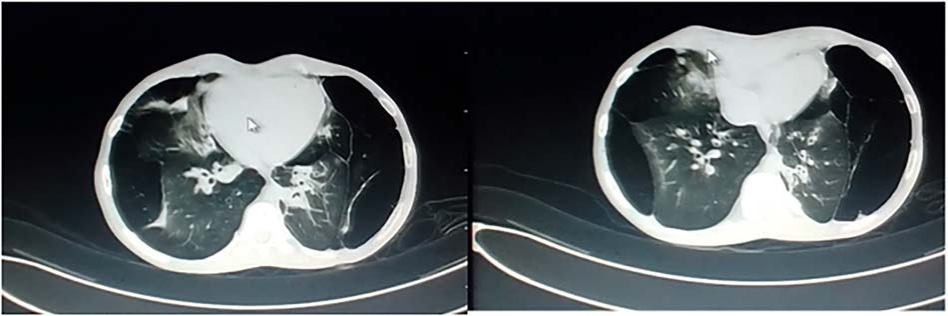
Axial view of CT scan of the chest showed multiple cystic lesions of varying sizes, with thin walls are seen in both lungs.

Chest X-ray was done and revealed a bilateral consolidated lung with reduced lung volume, bilateral area of lucency with devoid lung markings, cost-phrenic and cardio-phrenic angles were clear and mediastinum appeared normal. Diagnosis of bilateral pneumothorax was made with a differential diagnosis of giant bullae and chest computed tomography (CT) scan was recommended (Fig. 2). Spirometry was done and it revealed PEF reversibility of 20% after the administration of bronchodilator.

High-resolution CT scan showed multiple cystic lesions of varying sizes, with thin walls seen in both lungs, with the largest measuring: 10.89 × 9.79 × 7.45 cm in the upper lobe of the right lung. Associated consolidative collapse is seen in the right middle lobe. The trachea and both main bronchi have a normal course and caliber, and mediastinal structures, including the heart, are normal. Diagnosis of bullous emphysema was reached.

### Intraoperative findings

**Figure 3:**
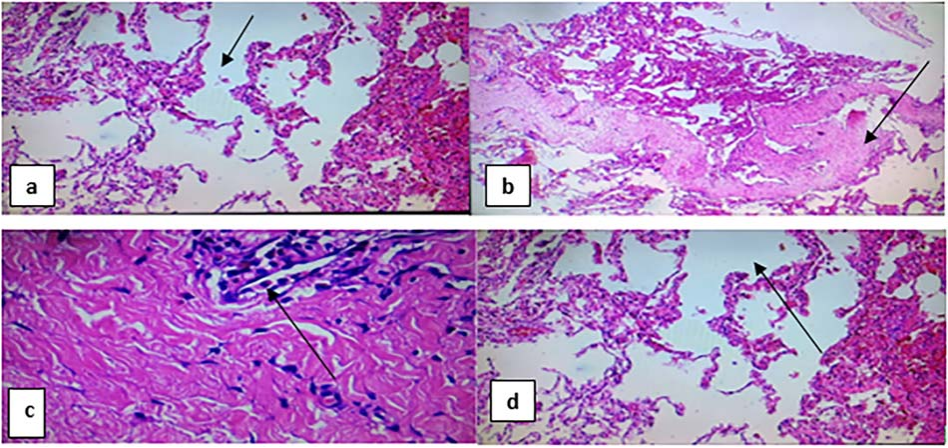
A huge bullae of the right hemi-thorax was seen; it was arising from right middle lobe.

Two-stage thoracotomy was performed first to access the right lung through a posterolateral approach. The chest showed emphysematous upper and middle lobes, while the lower lobe was normal. A bulla, occupying 50% of the right hemithorax, was observed arising from the right middle lobe (Fig. 3).

The patient was admitted to ICU, transferred to the surgical ward and received antibiotics, analgesics, fluid and daily physiotherapy with incentive spirometry. He was discharged home 10 days post-operation.

### Follow-up and outcome of interventions

**Figure 4:**
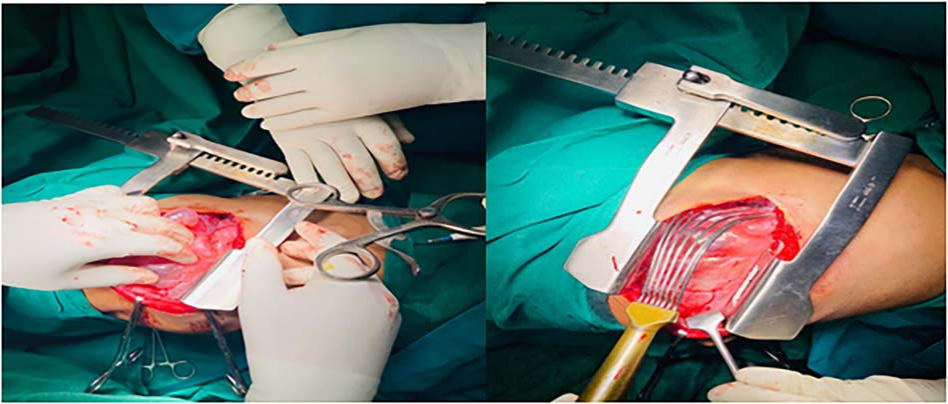
Section of tissue from the lung shows destructed alveoli, bronchiectasis organizing fibrosis (black arrow on **a** and **b**); large undulating cystic dilated alveoli with a fibrous wall and dilated vessels (black arrow on **d**); mild per-bronchial and parenchymal inflammatory cell infiltrate, lymphocytes and neutrophils (black arrow on **c**).

A left thoracotomy was done after 1 month through the same surgical approach; two large bullae located at the apices of the upper lobe and left lower lobe, heart, pericardium and the tracheobronchial tree were normal. Excision of the two bullae was done, and UWSD was applied and the chest was closed. The patient was admitted to ICU, then transferred to the ward and discharged after 7 days after he become became stable with improvement of pulmonary functions, and he was advised to follow-up. Tissue excised was sent for histology (Fig. 4), and it showed destructed alveoli, bronchiectasis and organizing fibrosis.

### Patient’s consent

The biological mother consented to the patient’s information being published, protecting the patient’s identity and ensuring their informed consent.

## DISCUSSION

Bronchial asthma patients experience chronic airway inflammation, which worsens as the condition progresses. Asthma symptoms include wheezing, shortness of breath, chest tightness, cough and fluctuating expiratory airflow limitation. These symptoms and airflow restrictions can change over time due to factors like exercise, allergies, weather changes and viral respiratory infections [5].

Bulla is an air-filled lung region with a diameter >1 cm, caused by emphysematous deterioration. Its pathophysiology involves chronic inflammation of distal airspaces, primarily caused by shocks like cigarette smoke, leading to alveolar wall breakdown and persistent airspace enlargement [6]. Smoking and airway inflammation contributes to asthmatic emphysema development, but asthmatics often display emphysema-like symptoms, and it is unclear whether asthma progresses to emphysema [7].

Asthmatics’ airway structural alterations can be detected through pulmonary function tests and irreversible airflow restriction, while chest plain films typically show normal results. Severe emphysema may be detected in 41% of patients using normal chest X-rays [8] However, A patient’s CXR revealed bilateral consolidated lung, reduced volume and bilateral area of lucency due to long-standing asthma episodes.

Over 30 years, emphysema surgery was primarily limited to giant bullous disease, with diffuse non-bullous resection considered a contraindication. Three main surgical procedures, giant bullectomy, LVRS and lung transplantation, are clinically effective for end-stage emphysema patients, each with unique physiologic entities, selection criteria and expected outcomes. Bullectomy is recommended for patients with massive bulla-like lung disease, while LVRS, lung transplantation or staged LVRS/lung transplant may be recommended for diffuse disease, depending on age, lung function, lobar predominance and unilateral or bilateral illness; however, the physiopathological basis of respiratory improvement after bullectomy and LVRS in patients with end-stage emphysema is the same [4].

The integrity of the lung’s inherent epithelial cell barrier is tightly linked to the pathophysiology of emphysema. When this barrier is irritated, by cigarette smoke, the inflammatory reaction delivers antigens to the bronchial-related lymphatic tissue layer. There, macrophages and neutrophils release enzymes such as elastase, which eventually destroys the lung’s epithelial lining [6].

## Data Availability

Not applicable.
